# Semi-Annual Meeting of the Medical Society of Erie County

**Published:** 1856-07

**Authors:** 


*Semi-Annual Meeting of the Medical Society of Erie County.*—The semi-annual meeting of the Medical Society of Erie County, was held in this city, on Tuesday, June 10th ult., at the room of the Buffalo Medical Association.

The attendance was unusually large.

The society was called to order about eleven o'clock, by the President, Dr. Wm. Van Pelt, of Williamsville.

After the reading and approval of the minutes, applications for membership with the society, were received from Drs. Whitehead, Ed. L. Holmes, George Hadley, and A. Jeyte, Jr. Their credentials having been reported as correct by an examining committee, they were severally elected members upon the condition of their complying with the various requisitions of the by-laws.

The committee appointed at the annual session to consider the suggestion contained in the President's Valedictory Address relative to the organization of a Society for the Relief of Distressed Members of the Profession, their Widows and Children, reported through Dr. Newman at length—endorsing the spirit of the address—detailing the manner of organizing—and leaving for the profession to decide upon its feasibility, as such decision involved the ability to decide upon the pecuniary means of the members of the profession; and concluded by offering the following resolutions:

*Resolved*, That the proposition of organizing a Society for the Relief of Widows and Orphans of Medical Men, as recommended in the Valedictory Address of the late President, is one which meets our hearty approval, and that we deem the subject worthy of an earnest effort to accomplish such an organization.

*Resolved*, That a committee be appointed to call together the members of the profession, at an early day, for the purpose of fully considering this subject, and taking such action thereon as may then be determined upon.

The report and resolutions were accepted and adopted, and a committee consisting of Drs. Flint, Wyckoff and Newman, was appointed.

A note was read from Dr. G. N. Burwell, the *orator* of the day, apologizing for his inability to comply with the duties of his appointment. The reasons given were deemed sufficient by the society, he was consequently excused, and was reäppointed to discharge that duty at the annual meeting in January next.

Dr. Wilcox offered the following resolution:

*Resolved*, That this society acknowledges the right, and under proper circumstances will exercise, discipline on its members for infraction of its rules.

Dr. White moved that this resolution be laid upon the table. He regarded its adoption an unnecessary repetition of action already frequently had by the society, and that it would be only a mere reäffirmation of what it has time and again declared as its right and rule of action.

The motion *to lie* upon the table, was adopted.

The society adjourned at noon to 2½ o'clock, P. M., when upon reässembling, Dr. Lockwood called up the following resolution offered by him Jan. 8th, 1856:

*Resolved*, That the fee-bill, relating to public appointments, adopted at the semi-annual meeting of this society in June, 1854, be, and the same is hereby repealed.

This resolution was debated *pro* and *con* at great length, and at the conclusion of the discussion, on motion of Dr. Wilcox, the *ayes* and *nays* were ordered taken, which was done with the following result: *Ayes* 7, *nays* 33, and the fee-bill consequently stands unrepealed.

Dr. Howell gave notice that at the next annual meeting he should move the following additional article to section 12th of the by-laws, to be known as Article 7, viz:

*Article* 7. Any regularly recognized physician may, on his introduction by a member of this association, and the consent of a majority of the association, be admitted to a seat at its meetings, EXCEPT such members as have been deemed worthy of expulsion, whose presence under any circumstances, until reädmitted by a four-fifth vote as provided by section 11 of article 7, shall not be allowed.

Dr. Hunt stated, that at a recent meeting of the Buffalo Medical Association, a committee had been appointed to take into consideration a project for furnishing the room of the association with medical periodicals, and of converting the same into a medical library and reading-room, and that the coöperation of this society was desired in the undertaking, and the consent to place the county library within the said room solicited, and suggested the propriety of appointing a committee to confer with the committee of the Buffalo Medical Association.

On motion of Dr. Ring, it was

*Resolved*, That a committee of three be appointed to confer with the Buffalo Medical Association upon this subject.

Drs. Treat, Gay, and Van Pelt, were appointed such committee.

The society adjourned to meet again Tuesday, July 8th, 1856.

---

The Medical Society of Monroe County, met at the City Hall, Rochester, on Wednesday, June 11th, 1856, the President, Dr. W. W. Ely in the chair. Dr. H. H. Langworthy, secretary.

Dr. Armstrong reported from a committee appointed to memorialize the Legislature.

A large number of cases of interest were then reported by Drs. McKay, John Reid, Armstrong, Reichenbach, Bangs, W. W. Reid, Sheman and Ely.

Dr. Armstrong read a dissertation on counterfeit members of the medical profession.

Drs. Thomas Arner and L. L. F. Hillman, were elected members.

Dr. Moore reported from committee on qualifications of students.

The president, Dr. W. W. Ely, then read his annual address "On the sugar-producing functions of the Liver," illustrating his subject by experiments.

The committee on officers nominated the following, who were duly elected by the society:

President—Dr. J. F. WHITBECK.

Vice-President—Dr. W. SLAYTON.

Treasurer—Dr. MOSES LONG.

Secretary—Dr. H. H. LANGWORTHY.

Censors—Drs. DEAN, MOORE, JOHN REID, MONTGOMERY, ARMSTRONG, ELY and BRIGGS.

Delegates to the State Medical Society—Drs. ARMSTRONG, W. W. REID, and EDSON.

The newly elected President, Dr. Whitbeck, took the chair.

On motion of Dr. Ely, a tax of $1.00 was voted on members.

On motion, Drs. Arner and John Reid were appointed to prepare dissertations for the next annual meeting.

The chair appointed as delegates to the National Medical Association, Drs. Moore, W. W. Reid, D. Carpenter, Montgomery, Avery and Langworthy.

On motion of Dr. Ely, six physicians were appointed to collect the names of all physicians living in the towns in this county, and report at the next annual meeting.

The chair appointed as such committee, Drs. T. V. B. Durand, J. Bangs, D. Carpenter, L. L. F. Hillman, Hazeltine and H. B. Miner.

*Resolved,* That this society having heard with deep regret of the death of one of our members, Dr. John D. Pillsbury, take this occasion to express our sorrow for the loss of a man who combined fine moral acquirements, with no common professional abilities, which well qualified him as an ornament, both to society and the medical profession.

---

*Allegany County Medical Society.*—Dr. C. M. Crandall, the President of this society, has issued a circular notifying all practitioners to apply for membership according to law, or subject themselves to the legal penalties for irregular practitioners. It may not be generally known that the county societies have the power to assess all practitioners, not members, one dollar per annum for the library fund of the society. This sum can be collected by suit in the name of the society.

*The Abortion Trade.*—There is something frightful in the perfect *sang-froid* with which women call upon the physician to relieve them from the duties of a mother. They walk into the office of our most honorable practitioners, state their case with the same ease of manner which would mark their description of a headache, and appeal for assistance with as perfect assurance of their right to it.

This sentiment is almost universal in the community, and it is nothing uncommon to see a most virtuous indignation on the part of the suffering female, as she listens to the refusal of her medical adviser to take any part in such an undertaking.

We have no space or disposition to discuss the morality of this infamous trade, but we do wish to enter our protest against those practitioners who accommodate themselves to this depraved moral sense of the female. We have an indirect personal knowledge of physicians who never refuse this kind of business; and whenever we procure any positive evidence we mean to use it against them. It is not our duty to act as a detective police, but there is room in this county for action in its medical societies with reference to some of their members; and we do not doubt that when the matter is brought up in proper shape, they will inflict the proper punishment without fear of consequences.

---

*Health of the Season.*—Here we are at the beginning of July, with a prospect of the continuance of that marked health in the community which has existed since the disappearance of the cholera in 1854. Since that time there has been no epidemic disease prevalent in Buffalo, and so far as we can judge of indications for the coming season, we are to be favored with another summer of unusual health. The weather has been cool and wet, the atmosphere lacking in humidity, and thus far we have had but few warm days. In April there was a general prevalence of diarrhœa in all parts of the city, which was thought by many to presage another cholera season, but it has entirely disappeared, and for a month past, physicians have had abundant leisure for fishing and hunting, or excursions into the country. We have chosen the latter form of amusement, and find in the sections visited a corresponding degree of health. Those country practitioners who have farms are engaged in planting corn, those who have not, in envying those who have.

*Ligature of the Arteria Innominata.*—We have received from the author, Prof. Paul F. Eve, of Nashville, Tenn., a pamphlet entitled "History of the Ligature applied to the Brachio-Cephalic Artery; with Statistics of the Operation."

Prof. Eve gives the history of all the recorded operations of ligating the innominata, commencing with Prof. Mott's operation—the first case undertaken—and including in all sixteen cases. Of these fifteen died from hæmorrhage, exhaustion or inflammation, at various periods varying from eleven hours to sixty-five days. The remaining case was that of Prof. Porter, of Dublin, who cut down upon the aneurismal sac, and found the innominata so much involved that he did not ligate, and singularly enough the operation resulted in a cure.

The result of Prof. Eve's paper is, that in all cases where the innominata has been ligated, the patient has succumbed, and that the operation is necessarily fatal. No one will again venture to perform it, and we may hope that it is now stricken out of the record of surgery. Yet any one, on reading the report of Prof. Mott's case, would feel that it furnished great encouragement for future operators. This encouragement no longer exists, for it is now evident that no surgical skill can avert a fatal result.

---

*New Journal.*—We have received the first number of the "Louisville Review and Bi-monthly Journal of Practical Medicine and Surgery." Edited by Prof. Gross and Dr. T. G. Richardson, of the University of Louisville.

We expected much from a journal thus conducted, and are happy to find our most flattering anticipations realised. The retrospective review of the works and cotemporaries of August Gottlieb Richter has all the brilliancy, sparkle and profundity of "Blackwood" in its best days. In the rapid improvement of American periodical literature it is no longer enough for an editor to be simply a sensible, practical man. The wants of a highly educated profession demand scholarship, combined with the qualities named. We have no room for further comment, but must express our gratification with everything about the "Louisville Review"—learning, brilliancy of style, high American tone, practical skill in choice of desirable matter, and, lastly, in beautiful mechanical execution.

